# Emerging roles of nucleotide metabolism in cancer development: progress and prospect

**DOI:** 10.18632/aging.202962

**Published:** 2021-05-05

**Authors:** Jingsong Ma, Mengya Zhong, Yubo Xiong, Zhi Gao, Zhengxin Wu, Yu Liu, Xuehui Hong

**Affiliations:** 1 Institute of Gastrointestinal Oncology, School of Medicine, Xiamen University, Fujian, Xiamen 361000, China; 2 Department of Gastrointestinal Surgery, Zhongshan Hospital, Xiamen University, Fujian, Xiamen 361000, China; 3 National Center for International Research of Biological Targeting Diagnosis and Therapy, Guangxi Key Laboratory of Biological Targeting Diagnosis and Therapy Research, Guangxi Medical University, Guangxi, Nanning 53000, China; 4 Medical College of Guangxi University, Guangxi, Nanning 530000, China; 5 General Surgery Center, Bazhong Central Hospital, Sichuan, Bazhong 636000, China

**Keywords:** nucleotide metabolism, tumor immunity, key metabolic enzyme, signaling pathway, oncogene-induced senescence

## Abstract

Abnormal cancer metabolism occurs throughout the development of tumors. Recent studies have shown that abnormal nucleotide metabolism not only accelerates the development of tumors but also inhibits the normal immune response in the tumor microenvironment. Although few relevant experiments and reports are available, study of the interaction between nucleotide metabolism and cancer development is rapidly developing. The intervention, alteration or regulation of molecular mechanisms related to abnormal nucleotide metabolism in tumor cells has become a new idea and strategy for the treatment of tumors and prevention of recurrence and metastasis. Determining how nucleotide metabolism regulates the occurrence and progression of tumors still needs long-term and extensive research and exploration.

## INTRODUCTION

Cancer cells utilized altered cellular metabolism to provide energy and biomacromolecules for self-renew and survival. Multiple metabolic processes have been proved to participate in cancer occurrence and progression [1, 2], among which nucleotide metabolism and the factors that influence this process are receiving increasing attention [3]. An in-depth understanding of this field is a prerequisite for the effective prevention and treatment of tumors, which is of great importance. The tumor incidence, detection rate and death rate are constantly increasing, and malignant tumors have become a common disease that seriously threatens human health [4]. The relationship between nucleotide metabolism and tumors has become a key studied issue [5, 6], but the specific molecular mechanism and function remain unclear. This review summarizes and discusses the above related research findings.

## Nucleotide metabolism

### Metabolism of purine nucleotides

The *de novo* synthesis of purine nucleotides is the main source of nucleotides *in vivo*, and the body mainly regulates the nucleotide synthesis rate through negative feedback [7]. Moreover, studies have shown that unlike nonproliferating cells, proliferating cells, such as immune cells and cancer cells, tend to use the *de novo* nucleotide synthesis pathway [3, 8]. Different tumor subtypes also differ in their choice of nucleotide synthesis pathway. For example, Martin et al. found that papillary breast cancer tends to utilize the *de novo* nucleotide synthesis pathway, while breast cancer with epithelial-mesenchymal transition characteristically preferentially the use of the salvage nucleotide synthesis pathway [9].

Yamaoka et al. suggested that the formation of 5′-phosphoribose-1′-pyrophosphate (PRPP) and 5′-phosphoribosamine (PRA) is the main regulatory process in this pathway and that 5′-phosphoribosyl-1′-pyrophosphate synthetase (PRS) and glutamine phosphoribosylpyrophosphate amidotransferase (GPRATase), which catalyze the two reactions, are the key enzymes that are regulated in the pathway [10, 11].

In addition, during the synthesis of AMP and CMP from IMP, hypoxanthine nucleotide dehydrogenase (IMPDH) and adenylosuccinate synthetase (ADSS) are the key enzymes in each synthetic route [12, 13].

The purine nucleotide salvage pathway is simpler and less energy-consuming than the *de novo* synthetic approach [14, 15], and its relative importance depends on the synthesis conditions and specific tissue type. Some tissues and organs in the body, such as the brain and bone marrow, lack the enzyme system necessary to synthesize purine nucleotides from scratch, so they can utilize only the salvage approach to synthesize purines [16–18].

### Metabolism of pyrimidine nucleotides

The synthesis of pyrimidine nucleotides, like that of purine nucleotides, takes simple substances such as CO_2_ and glutamine as raw materials and is also divided into *de novo* synthesis and salvage pathways [19]. PRPP is at the intersection of two synthetic pathways and involved in both [20].

In mammalian cells, the key enzymes in the synthesis of pyrimidine nucleotides are carbamyl phosphate synthetase II (CPSII) and dihydroorotate dehydrogenase (DHODH), which are regulated by a UMP negative feedback mechanism [21, 22].

## Nucleotides and tumors

As a kind of biological information macromolecule, nucleotides mainly function as the raw materials for nucleic acid synthesis to support cell proliferation [23, 24]. With continuous in-depth study of purines and pyrimidines, our understanding of nucleotides in tumors has revealed their nonproliferative effect beyond their effect on cell growth [25, 26]. The role of purine molecules as purinergic signaling ligands has been fully recognized [27, 28]. Recently, great progress has been made in understanding the nonproliferative role of pyrimidine molecules. Aarif et al. found in a breast cancer model that after the thymidylate synthase (TS) gene was knocked out, a gene characteristic of epithelial-mesenchymal transition (EMT) in tumors was inhibited, and TS-deficient cells showed decreased invasion and metastasis *in vivo* [29]. Many researchers are committed to providing pioneering ideas for increased understanding and the prevention of tumors.

### Nucleotide metabolism and tumor immunity

The immune microenvironment is an important part of the tumor microenvironment [30], and the relationship between nucleotide metabolism in tumor cells and immune cells is emerging [31]. Cancer cells, virus-infected cells, cells that undergo rapid proliferation and other abnormal cell types express the cell-surface glycoprotein MHC class I polypeptide-related sequence A (MICA), which can be identified by Natural killer group 2D (NKG2D), allowing immune identification and the removal of potential pathological cells [32]. The Michael team found that glucose transport to cells and active glycolytic metabolism are necessary to increase the expression of MICA, and purine synthesis is necessary to support this effect of glucose. An increase in purine nucleotide levels is sufficient to induce the expression of MICA and acts as the core component of MICA induction [33].

In the pathogenic process of tumors, under metabolic stress or hypoxia, tumors and immune cells produce adenosine, which decomposes into precursor purine nucleotides [34, 35]. Adenosine receptors (A1, A2A, A2B and A3) are found on the surface of various immune cells [36–39]. Studies have shown that adenosine acts as a reporter, reducing inflammatory immune signals by binding adenosine receptors [40–42]. In 1999, Xaus et al. found that macrophages express all four adenosine receptors. Adenosine prevents monocytes from dividing into macrophages and inhibits the proliferation of murine bone marrow-derived macrophages, which relies on macrophage colony stimulating factor (M-CSF) [43]. With further exploration, researchers found that adenosine interacts to varying degrees with different types of immune cells in tumor tissues; in fully mature dendritic cells, adenosine strongly inhibits the release of Interleukin-12 (IL-12) induced by Toll-like receptors (TLRs) by binding A2A receptors and inhibits the antitumor immune response. IL-12 is a strong antitumor cytokine, and the inhibitory effect of adenosine on IL-12 release promotes tumor growth [44]. Furthermore, an increase in adenosine levels in the tumor environment inhibits the lytic activity of natural killer cells by the binding of adenosine to A2A receptors [45]. Adenosine also inhibits the release of various immunomodulatory cytokines in the T cell-mediated adaptive immune response by binding the A2A and A2B receptors [46]. Recent reports indicate that adenosine also plays an equally important role in the immune suppression of regulatory T cells [47–49]. The relationship between nucleotide metabolism and tumor immunity is shown in Table 1.

**Table 1:** Summary of the relationship between nucleotide metabolism and tumor immunity.

**Nucleotide**	**Targets**	**Immune cells**
Purines	MICA	Natural killer cells
	A1, A2A, A2B and A3	Monocytes, Macrophages
	A2A	Dendritic cells
	A2A	Natural killer cells
	A2A, A2B	T cells, Regulatory T cells

As mentioned above, nucleosides are decomposed from nucleotide acids [50], and nucleotide acids and their decomposition products, nucleosides, have diverse effects. For example, ATP and ADP have immunostimulatory functions and can stimulate natural killer cells in the spleen to absorb antigens [51]. The immunosuppressive function of nucleosides, including adenosine, is very extensive. All immune cells express receptors for extracellular nucleosides and nucleotide acids (such as adenosine and ATP) [52]. Pyrimidine nucleotides show selective affinity for certain receptor subtypes; therefore, blocking extracellular nucleotide metabolism to restore tumor immunotherapy interventions has provided new ideas and insight into the development of new antitumor small-molecule drugs targeting nucleotide metabolism [53, 54].

### Late potential for the development of new antitumor drugs targeting organ-specific nucleotide metabolases in tumors

Nucleotide metabolism is the final and most critical link in tumor cell replication [55]. Tumor cells synthesize DNA and RNA through nucleotide metabolism to achieve uncontrolled self-proliferation [2].

In recent years, although nucleotide synthesis metabolic pathways, especially their importance and function, have attracted increasing attention, the key molecules and regulatory mechanisms involved in nucleotide metabolism are not very clear. All classical antitumor drugs, such as methotrexate and 5-fluorouracil [56, 57], are based on analogs of tumor nucleotide metabolites. However, due to their lack of specificity for tumor cell nucleotide metabolism, these drugs also inhibit the metabolic processes of normal cells, causing serious side effects [58–60]. Therefore, more in-depth study of the regulatory processes of nucleotide metabolism has very important theoretical and clinical significance.

At present, research on the related enzymes that regulate nucleotide metabolism in tumor cells is relatively scarce [61]. Hong et al. found that the nucleotide metabolism of digestive tract tumor cells varies in different diseased organs of the digestive tract, showing obvious organ specificity. By conducted more in-depth research using digestive tract tumors from different pathogenic organs as models, a specific key kinase that regulates the rate-limiting enzyme activity of nucleotide metabolism was discovered; in research on nucleotide synthesis and metabolism in gastric cancer, the kinase UHMK1 involved in the nucleotide anabolism of gastric cancer was found to activate the *de novo* rate-limiting purine anabolism-related enzymes 5′-aminoimidazole-4′-carboxamide ribonucleotide formyltransferase (ATIC) and inosine monophosphate dehydrogenase (IMPDH) by regulating the NCOA3/ATF4 axis, promoting the occurrence and development of gastric cancer [62]. In research on nucleotide anabolism in cholangiocarcinoma, CDC like kinase 3 (CLK3) was found to activate the rate-limiting enzyme in *de novo* purine anabolism, ATIC, by regulating the USP13/Fbxl14/c-Myc signaling axis, thereby promoting the molecular progression of cholangiocarcinoma. Furthermore, through large-scale small-molecule drug screening, tacrine hydrochloride was found to target CLK3 to treat cholangiocarcinoma, reducing the cholangiocarcinoma tumor formation rate by 85% and reducing the nucleotide level. Further understanding of tumor cell DNA repair is expected to provide a new strategy for the combined treatment of clinical cholangiocarcinoma [63]. In research on nucleotide anabolism in hepatocellular carcinoma, dual-specificity tyrosine phosphorylation-regulated kinase 3 (Dyrk3) was found to limit *de novo* purine anabolism by regulating the transcriptional activity of ATF4 and inhibiting the rate-limiting *de novo* purine anabolism-related enzyme 5′-phosphoribosyl pyrophosphate amidotransferase (PPAT), thereby inhibiting the growth and metastasis of hepatocellular carcinoma [64]. Information on organ-specific nucleotide metabolases in tumors are shown in Table 2.

**Table 2:** Organ-specific nucleotide metabolases in tumors.

**Tumor/cell type**	**Kinase**	**Signaling axis**	**Enzymes**
Gastric cancer	UHMK1	NCOA3/ATF4	ATIC, IMPDH
Cholangiocarcinoma	CLK3	USP13/Fbxl14/c-Myc	ATIC
Hepatocellular carcinoma	Dyrk3	NCOA3/ATF4	PPAT
Glioblastoma-initiating cells			DHODH

Smile et al. recently conducted high-throughput drug screening on a Fuji film library containing 10,560 compounds to identify drugs to eradicate glioblastoma-initiating cells (GICs). After screening the library layer by layer, seven compounds displaying 50% growth inhibition at a concentration less than 1 μM were identified, and two compounds with similar structures, 9700 and 10607, were ultimately identified from the seven compounds. Through mass spectrometry and *in vitro* enzyme activity inhibition experiments, the target protein of the above compounds was confirmed to be DHODH, and the effect of 10607 was obviously stronger than that of 9700, but its stability was lower. The researchers screened compound 10580, which exhibits high stability, based on its chemical type. Both compounds 10607 and 10580 contain 2′-amino-5′-cyclopropyl nicotinic acid and indole structural regions, which are not possessed by traditional DHODH inhibitors such as leflunomide and teriflunomide [65].

These research results have not only greatly enriched understanding of the regulatory mechanism of tumor cell nucleotide metabolism but also provided insights into the clinical development of new specific therapeutic drugs. Large-scale screening of small-molecule compounds targeting the above kinases, *in vivo* and *in vitro* antitumor pharmacodynamics experiments and in-depth studies of molecular mechanism are expected to overcome the deficiencies of existing drugs [66, 67].

### Oncogenes and tumor-suppressor genes regulate tumor nucleotide metabolism through signaling pathways

Based on the impact of an increasing number of new ideas, such as information on gene mutations and immune escape, the notion that pathological metabolism in tumors occurs through the “Warburg effect” has been gradually disregarded [68–70]. At present, an increasing number of studies have shown that oncogenes and tumor-suppressor genes are key regulatory molecules in *de novo* nucleotide synthesis and that changes in these genes regulate the growth and metabolism of tumor cells through specific signaling pathways [71].

Karina et al. found that the absence of sirtuin 3 (SIRT3) could enhance mechanical target of rapamycin complex 1 (mTOCR1) signal transduction, thus significantly upregulating the transfer of glutamine to the nucleotide metabolism pathway [72]. Naiara et al. found in an experimental model of pancreatic ductal adenocarcinoma (PDAC) that the proto-oncogene K-RAS could activate mitogen-activated protein kinase (MAPK), leading to an increase in the expression of the oncogene MYC and finally increasing the transcriptional activity of ribose 5′-phosphate isomerase A (RPIA), a raw material necessary for nucleotide metabolism, in the nonoxidative pentose phosphate pathway (PPP) [73]. That is, the proto-oncogene K-RAS enhanced the new synthesis of purine and pyrimidine in PDAC by upregulating the transcriptional activation of RPIA mediated by MYC and maintained high nucleotide levels in cells [74].

P53 is an important tumor suppressor gene. Mutant P53 (mtP53) has been proven to promote the occurrence and development of tumors [75–78]. Martinez et al. confirmed that mtP53 is related to the promoters of numerous nucleotide metabolism genes (NMGs), which promote the biosynthesis of nucleotides by upregulating NMGs at the transcriptional level. Experiments have shown that an ETS-binding site is present in the NMG promoter and that ETS proto-oncogene 2 (ETS2) can recruit mtP53 to the promoter region containing the ETS-binding site [79]; furthermore, the synergistic stimulation of both increased the expression of NMG, thus exerting metabolic activity to drive and maintain tumor occurrence and development [80].

An increasingly deep understanding of how oncogenes and tumor-suppressor genes regulate tumor nucleotide metabolism through signaling pathways provides hope for reasonable, metabolism-oriented cancer therapy, which must be based on a comprehensive understanding of host and tumor metabolism.

### Nucleotide metabolism and oncogene-induced senescence

The Katherine team proved that senescence is a form of stable cell growth arrest. When activated oncogenes (RAS, BRAF, etc.) are expressed in normal cells, the cells produce abnormal proliferation signals, placing the cells in a state of growth arrest and inhibiting proliferation of the cells [5, 81]. Senescence induced by oncogenes has been recognized as a true anticancer mechanism [82–85]. Studies have shown that in the process of oncogene-induced senescence, nucleotide metabolism pathways are generally downregulated. The occurrence of OIS requires almost all deoxyribonucleoside triphosphates to be exhausted and DNA replication to be activated [86].

As discussed above, nucleotide metabolism plays an important role in tumor formation and progression. Moreover, the expression of ribonucleotide reductase regulatory subunit M2 (RRM2) is tumorigenic [87], and the nucleotide-metabolizing enzyme thymine synthase (TS) can independently transform cells in the body and cause tumor formation [88]. Thus, we can use components of the nucleotide metabolism pathways, such as RRM2 or TS, as diagnostic and prognostic biomarkers for a variety of tumors [89].

## Summary

With increasingly in-depth research, the relationship between nucleotide metabolism, tumor occurrence and development and the immune microenvironment has become increasingly clear [90]. Accordingly, determining how tumor progression can be inhibited by interfering with nucleotide metabolism has received increasing attention. We need to fully understand that nucleotide metabolism is a complex process involving multiple catalytic enzymes, and an accurate understanding of this process will be beneficial to the research and development of tumor-specific drugs, improving the survival and prognosis of tumor patients. At present, there has been relatively little research in this field, and further research is needed to reveal the relationship between nucleotide metabolism and tumors. Exploring and clarifying this complex mechanism will become a hot research direction in the future.
